# Identification of circRNAs in industrial hemp (*Cannabis sativa* L.) and prediction of regulatory networks underlying female differentiation

**DOI:** 10.3389/fpls.2026.1856539

**Published:** 2026-08-10

**Authors:** Qiang Wang, Zhibo Sun, Nan Zhao, Xinyue Wang, Lijie Liu, Xuesong Wang

**Affiliations:** 1 College of Life Science and Agriculture and Forestry, Qiqihar University, Qiqihar, Heilongjiang, China; 2 Engineering Research Center for Industrial Hemp and Industrial Hemp Products in Cold Regions, Ministry of Education, Qiqihar, Heilongjiang, China

**Keywords:** circRNA, circRNA identification, female sex differentiation, industrial hemp (*Cannabis sativa* L.), miRNA

## Abstract

**Introduction:**

Circular RNAs (circRNAs) are important regulators of plant development and stress responses, but their roles in 6-benzylaminopurine (6-BA)-induced female flower differentiation in industrial hemp remain unclear.

**Methods:**

Industrial hemp plants were treated with 60 mg·L-1 6-BA, and female flower samples were collected at different developmental stages. Genome-wide high-throughput sequencing was used to identify circRNAs. Selected circRNAs and miRNAs were validated by qRT-PCR. Gene Ontology and Kyoto Encyclopedia of Genes and Genomes analyses were performed, and putative circRNA–miRNA–mRNA networks and the protein-coding potential of circRNAs were computationally evaluated.

**Results:**

A total of 264 circRNAs were identified. Multiple circRNAs exhibited stage-specific responses to 6-BA. Cs_circ_67024327 showed the strongest induction at the early female flower stage, whereas Cs_circ_44508988 and Cs_circ_21994222 exhibited distinct responses from the early to middle developmental stages. CsSPT5, a flowering-time candidate gene located near the SDR/PAR boundary, was downregulated at the shoot apical meristem stage but upregulated at the early and middle female flower stages. No statistically significant GO terms or KEGG pathways were detected after multiple-testing correction. Computational analyses suggested that several circRNAs may function as miRNA sponges, and some circRNAs contained conserved domains indicating potential protein-coding capacity.

**Discussion:**

These findings provide preliminary evidence that 6-BA-responsive circRNAs may participate in female flower differentiation in industrial hemp through predicted ceRNA interactions or mechanisms related to their coding potential. These putative regulatory mechanisms require further experimental validation.

## 1 Introduction

Industrial hemp (*Cannabis sativa* L.) is an annual herbaceous plant of the family Cannabaceae. Industrial hemp, also known as Hanma, is defined as *Cannabis* cultivars with a tetrahydrocannabinol (THC) content of less than 0.3% (dry weight) (Sun, 2023). Industrial hemp is predominantly dioecious, with only a small proportion of cultivars monoecious (Malabadi et al., 2023), and as an economically important crop, its sex differentiation directly affects fibre yield and seed quality. Female flower differentiation is a key process for high yield in industrial hemp. Although exogenous plant growth regulators such as 6-benzylaminopurine (6-BA) significantly promote female flower formation (Yu et al., 2024), the underlying molecular mechanisms, especially the regulatory roles of long non-coding RNAs (lncRNA), remain largely elusive. As a cytokinin, 6-BA modulates floral organ development by regulating endogenous hormone homeostasis and signal transduction pathways (Aremu et al., 2020). With the rapid development of molecular biology and diverse bioinformatic, molecular studies on industrial hemp have been extensively conducted. It is reported that the first industrial hemp pangenome was conducted, revealing that the sex-determining region (SDR) contains 840-1,160 genes, of which ~6% are directly associated with male development. They further identified polymorphism in the Y-chromosome-linked flowering-time gene *SPT5*, whose differential expression may affect the stability of sex differentiation (Lynch et al., 2025). Recently, multi-omics analyses of the industrial hemp genome in lncRNA have identified extensive alternative splicing events and revealed the regulatory roles of lncRNAs and microRNAs (miRNAs) in the cannabinoid and cellulose biosynthesis (Wu et al., 2021).

CircRNAs are a distinct class of RNA molecules with covalently closed circular structures. Lacking a 5’ cap and 3’ poly(A) tail, they are resistant to exonuclease degradation and thus exhibit superior stability (Chu et al., 2017). Compared with linear RNAs, circRNAs are more evolutionarily conserved, and their expression exhibits tissue specificity and developmental stage dependence (Zhang et al., 2020), playing critical roles in plant growth, development and stress responses. Early studies have demonstrated that circRNAs regulate gene expression in animals through diverse mechanisms. For example, circRNAs can function as competing endogenous RNAs (ceRNAs) by binding miRNAs to alleviate miRNA-mediated repression of target genes, thereby establishing circRNA-miRNA-mRNA regulatory networks (Ghafouri et al., 2024). Although research on plant circRNAs started relatively late, recent studies have gradually revealed their diverse functional roles in plants. A subset of plant circRNAs has been reported to encode functional peptides involved in stress responses, while others act as miRNA sponges and play crucial roles in processes including floral organ development (Waheed and Zeng, 2020) and fruit ripening (Zuo et al., 2019). For example, a circRNA derived from the intron of *At5g37720* significantly delays flowering in *Arabidopsis thaliana* (Cheng et al., 2018), whereas in tomato, circRNAs modulate fruit ripening by interacting with *miR164* (Lin et al., 2022). Collectively, these findings demonstrate that circRNAs are indispensable components of gene expression regulatory network in plants.

While research on plant circRNAs has advanced in recent years, with several circRNAs already identified in industrial hemp, the specific functions of circRNAs in 6-BA treated industrial hemp female flowers remain largely elusive. In this study, female flowers of industrial hemp treated with 60 mg·L^-1^ 6-BA were subjected to high-throughput RNA sequencing (RNA-seq) to systematically identify circRNAs responsive to 6-BA treatment. Additionally, we analysed the expression dynamics of circRNA across different developmental stages of industrial hemp female flowers, performed functional enrichment analysis of their parental genes, and predicted a putative circRNA-miRNA-mRNA regulatory network. We further explored the potential of circRNAs to act as miRNA sponges and evaluated their protein-coding potential. These findings elucidate the molecular mechanisms underlying 6-BA-induced female flower differentiation in industrial hemp, offer novel insights into the regulatory roles of circRNAs in plant sex determination, and provide a new theoretical basis and potential targets for the molecular breeding of industrial hemp sex differentiation.

## 2 Materials and methods

### 2.1 Plant materials and treatments

The industrial hemp cultivar ‘Longma 5’ was used in this study. Large, plump seeds were selected and soaked in distilled water for 72 h. Seeds were then sown in seedling pots containing a 3:1 mixture of loose, fertile horticultural soil and vermiculite, and allowed to germinate under natural light at 25°C. When industrial hemp seedlings reached the three-leaf stage, 6-BA solution at a concentration of 60 mg·L^-^¹ was sprayed onto the shoot apical meristem (SAM) of seedlings in the treatment group. Each spray was terminated once the leaf surface was fully wetted and droplets started to run off. The application was performed once every three days for a total of three times. The treatments were applied on days 0, 3, and 6. Seedlings in the control group were sprayed with distilled water following the same regimen. At the SAM stage of industrial hemp, the vegetative meristem appears as a light-green conical structure surrounded by leaf primordia. We classified the developmental stages of female flowers mainly based on changes in inflorescence morphology and stigma color. At the early stage, the stigmas were white or pale, fresh and erect, with small, tightly enclosing bracts and a loosely arranged inflorescence. At the middle stage, the stigmas began to turn reddish-brown, the bracts became swollen, and glandular trichomes (appearing as white villi) became more abundant. At the late stage of female flower differentiation, the stigmas turned dark brown and appeared withered and curled. Identical staging criteria were applied to both the control group and the 6-BA treatment group. Based on the above morphological characteristics of the SAM and the established staging criteria for female flower differentiation, we separately harvested female flower samples at each developmental stage from both the control and treatment groups. For each of the control and treatment groups, 15 plants were randomly selected. Female flower samples from five plants were pooled as one biological replicate, and three biological replicates were prepared for each group. All samples were rapidly frozen in liquid nitrogen, stored at -80 °C, and subsequently subjected to high-throughput sequencing analysis.

### 2.2 Total RNA and genomic DNA extraction from industrial hemp

Total RNA was isolated from female industrial hemp flowers at different developmental stages using the TRIzol Universal Plant RNA Extraction Kit (TIANGEN, Beijing, China) following the manufacturer’s protocol. RNA concentration and purity were measured with a NanoDrop 2000 spectrophotometer, and RNA integrity was verified by agarose gel electrophoresis. Genomic DNA was isolated from female industrial hemp flowers at different developmental stages using the Ezup Column Plant Genomic DNA Extraction Kit (Sangon Biotech, Shanghai, China) following the manufacturer’s protocol, and was used as a negative control for divergent primer amplification.

### 2.3 CircRNA library preparation and whole-transcriptome sequencing

Sequencing libraries were prepared using a strand-specific rRNA depletion protocol and subjected to circRNA sequencing. Strand-specific libraries were prepared using the Illumina^®^ Stranded Total RNA Prep, Ligation with Ribo-Zero Plus, and Microbiome kit. Total RNA was extracted from tissue samples, and RNA concentration and purity were measured with a NanoDrop 2000 spectrophotometer. RNA integrity was verified by agarose gel electrophoresis, and RNA quality was further assessed by determining the RNA quality number (RQN) with an Agilent 5300 system. For library preparation, each sample was required to contain 1 μg of total RNA, with a concentration ≥100 ng·μL^-1^, an RQN >6.5, and an OD260/280 ratio of 1.8-2.2. RNA-seq libraries were prepared using rRNA-depleted RNA. First-strand cDNA was synthesized from mRNA templates using random primers and reverse transcriptase. During second-strand synthesis, dUTP was substituted for dTTP in the dNTP mixture, such that the second cDNA strand contained A, U, C and G bases. A single adenine (A) was added to the 3’ ends of the double-stranded cDNA to enable subsequent adaptor ligation. Before PCR amplification, the second cDNA strand was selectively degraded using uracil-N-glycosylase (UNG), ensuring that only the first cDNA strand was retained in the final library. The resulting libraries were subsequently amplified by PCR and purified to generate the final sequencing libraries. The purified libraries were subjected to high-throughput sequencing on the Illumina NovaSeq X Plus platform.

### 2.4 Computational identification of circRNAs

Raw sequencing data were first subjected to quality filtering to generate high-quality clean reads for downstream analysis. The filtered reads were then mapped to the reference genome (GCF_900626175.2), and the resulting alignments were used for subsequent transcript assembly and expression quantification. The mapping results were further evaluated to assess the quality of the transcriptome sequencing data. Putative circRNAs were identified from back-splice junction (BSJ) reads using CIRI2. After gene-level read counts were obtained, differential expression analysis was conducted for multi-sample datasets (≥2 samples) to identify differentially expressed circRNAs (DECs), which were then subjected to downstream functional analysis. For datasets with biological replicates, differential expression analysis was carried out using DESeq2. CircRNAs meeting the thresholds of FDR<0.05 and |log_2_FC|≥1 were considered DECs.

### 2.5 Validation of industrial hemp circRNAs

To validate the reliability of circRNA identification results, 19 representative DECs were selected for experimental verification (**Supplementary Table 1**). Selection was based on comprehensive criteria including expression abundance, fold change, genomic origins (exonic, intronic,intergenic), and expression trends (up- and down-regulated), ensuring representative validation coverage. Divergent primers spanning the back-splice junctions were designed to amplify the head-to-tail junctions of the circular RNA templates. Divergent and convergent primers were designed from the circular and corresponding linear sequences of the circRNAs, respectively (**Table 1**). Genomic DNA and cDNA served as templates for PCR amplification using the convergent and divergent primer sets. PCR was performed with an initial denaturation at 94 °C for 5 min, followed by 42 cycles of denaturation at 94 °C for 30 s, annealing for 30 s at the primer-specific temperature, and extension at 72 °C for 30 s, and a final extension at 72 °C for 10 min. After verification by agarose gel electrophoresis (AGE), PCR products were purified using a gel extraction kit (Sangon Biotech, Shanghai, China). Purified PCR products were ligated into a vector, transformed into competent cells, and subjected to positive clone screening. The PCR products were then validated by Sanger sequencing to confirm the presence of the predicted back-splice junctions of circRNAs in industrial hemp.

**Table 1:** Primers of back-splicing sites used in verification of circRNAs.

Type of primers	Name of primers	Sequence of primers (5’-3’)
divergent primers	*Cs_circ_21994222*-F	AGACAAAGGGTTGCAACCGTG
	*Cs_circ_21994222*-R	CGAACTTTGACCCCCATGTTG
	*Cs_circ_67024327*-F	AGGGTTGTTGGAGGAACAAGAGG
	*Cs_circ_67024327*-R	TTCAAGGCCAAGAAACTGCCC
	*Cs_circ_13924653*-F	CATCACACCAGGACCTCTCTGAATT
	*Cs_circ_13924653*-R	CGTGCTTCATCATCATCATCATCTC
	*Cs_circ_44508988*-F	GTCTGACAGGAGGTATTCCCAATGA
	*Cs_circ_44508988*-R	TTGTTAGCCACCAAGTTCAGTTGTG
	*Cs_circ_62648206*-F	AGTACAAGGAGTGCAGCAAAGGC
	*Cs_circ_62648206*-R	GCAACCAGAGAGAAAGCCCAAT
	*Cs_circ_7359214*-F	ACGTTGGTGTCATCAGAGCTGG
	*Cs_circ_7359214*-R	GAAGATCCTCTTCCAGGGTTGC
	*Cs_circ_78453652*-F	AATCATGATGGTGTTGAGAAAGTGG
	*Cs_circ_78453652*-R	AACTTGTTTCCACTTCCCACTTTCT
	*Cs_circ_10174711*-F	AACATTGGCTGGTCAAGAGTTATCA
	*Cs_circ_10174711*-R	CTTCCAGAGTACGTTGAAAAAGCTG
	*Cs_circ_93950065*-F	ATTTCACTCCATTACCACTCTTCCG
	*Cs_circ_93950065*-R	TAACAGCACCAACATCACCAAAATC
	*Cs_circ_26089176*-F	TGGTAATGATCAGTCACCCTATGGA
	*Cs_circ_26089176*-R	GTTTGGTTGGTTTTACACCCAGTTT
	*Cs_circ_68328938*-F	TGGACGATGATAGTTTCATGTATGC
	*Cs_circ_68328938*-R	CGATTTCAATCAGGTCGTCATAAG
	*Cs_circ_78297088*-F	AAACCGGAAGGCTTCTTATTAGTCA
	*Cs_circ_78297088*-R	CATTACAGATAACTGAGCACCCCAA
	*Cs_circ_1525928*-F	TGAGATTGAGAGATTGGTTATGGGT
	*Cs_circ_1525928*-R	GAACAGTCATACGGTTTCTTACCGT
	*Cs_circ_64202677*-F	CTTCCAGCCATCTCAATTAAGGATG
	*Cs_circ_64202677*-R	CAGCTAAGAATAGCTCCTCCAAACG
	*Cs_circ_52541209*-F	ACTCAGCTAGTGGAGGATTCGATGA
	*Cs_circ_52541209*-R	TCAATTAGCGCCTCTGGATACTCTT
	*Cs_circ_41570886*-F	AAGGAAGGAGACACAGTTCTTTTGC
	*Cs_circ_41570886*-R	AGTTTCCCATCTCTATCACGACCTC
	*Cs_circ_93504887*-F	TATGAATCGGATTATGCTGAGGACC
	*Cs_circ_93504887*-R	ACACGTCAGATTTGAGGGAGAATTG
	*Cs_circ_59811684*-F	TCAAACTTTTAGAGGAACATGGTGC
	*Cs_circ_59811684*-R	TCACTTCTTCATTTTTGTAATGCACAG
	*Cs_circ_62648012*-F	ACACATTGAGGCCAAAGACAAAAAG
	*Cs_circ_62648012*-R	ACTAACTTCATCAGAGCTGCCCAGA
convergent primers	*Cs_circ_41570886*-F	AAGGAAGGAGACACAGTTCTTTTGC
	*Cs_circ_41570886*-R	AGTTTCCCATCTCTATCACGACCTC
	*Cs_circ_93504887*-F	TATGAATCGGATTATGCTGAGGACC
	*Cs_circ_93504887*-R	ACACGTCAGATTTGAGGGAGAATTG
	*Cs_circ_21994222*-F	GGCTGAATGAAGGGTTTGCAA
	*Cs_circ_21994222*-R	TCCATACATTCCATTCCGGGA
	*Cs_circ_67024327*-F	TGAACAGTGGCAGGCCTAGGA
	*Cs_circ_67024327*-R	TCCTCCACCGAGAACACATGC
	*Cs_circ_13924653*-F	CAGGTACTTCACTCGAAAAGCCA
	*Cs_circ_13924653*-R	CTCCAATCCAAACAAACAAGGG
	*Cs_circ_44508988*-F	TCAGGGCCTTTACCGAGATGG
	*Cs_circ_44508988*-R	TGAAGGGGCTTACGAATTGGG
	*Cs_circ_62648206*-F	TTGCCTGAATTAGTCCCCGTG
	*Cs_circ_62648206*-R	TGAAGGCTTGAACCTGGCTTG

### 2.6 Annotation and functional enrichment analysis of host genes of DECs and target genes of differentially expressed miRNAs under 6-BA treatment

To investigate the potential biological functions of host genes of DECs and target genes of differentially expressed miRNAs (DEMs) under 6-BA treatment, GO and KEGG enrichment analyses were performed separately for the two gene sets. For GO enrichment analysis, the candidate genes were mapped to the GO database and classified into biological process, cellular component, and molecular function categories. Enrichment analysis was conducted using the hypergeometric test, with all annotated genes in the industrial hemp genome used as the background gene set. For KEGG pathway enrichment analysis, the candidate genes were annotated against the KEGG database, and enrichment analysis was performed using the default statistical method of the enrichment analysis platform. *P-*values were corrected for multiple testing using the Benjamini-Hochberg method. Adjusted *P*-value (Padjust) < 0.05 were considered significantly enriched, while terms with a raw *P*-value < 0.05 but Padjust ≥ 0.05 were regarded as showing potential functional trends rather than statistically significant enrichment. DEMs were identified by DESeq2, with FDR < 0.05 and |log_2_FC| ≥ 1. Subsequently, target prediction was performed for all known and novel miRNAs using the plant-specific tool psRobot, to predict putative targets among mRNAs, lncRNAs, and circRNAs, and to infer their potential regulatory interactions with miRNAs.

### 2.7 Differential expression analysis of circRNAs, miRNAs and *CsSPT5* in industrial hemp under 6-BA treatment

To validate the expression profiles of DECs and DEMs identified by RNA-seq, as well as those of the known cannabis sex-associated *CsSPT5*, qRT-PCR was performed on selected circRNAs, miRNAs and *CsSPT5*. Female flower samples were collected at the SAM stage, early-, mid- and late-flowering under 60 mg·L^-1^ 6-BA treatment, with corresponding samples from distilled water-treated controls collected at the same developmental stages for comparison. Primer sequences of DECs and *CsSPT5* are provided (**Table 2**). qRT-PCR was conducted using TB Green^®^ Premix Ex Taq™ (Tli RNaseH Plus) (TaKaRa, Beijing, China). MiRNA-specific forward primers were designed for each target (**Table 3**), and qRT-PCR analysis was conducted using the Mir-X™ miRNA First-Strand Synthesis Kit and TB Green^®^ qRT-PCR reagents (TaKaRa, Beijing, China).

**Table 2:** Primers used in quantitative real-time PCR.

Name of primers	Sequence of primers (5’-3’)
*Cs_circ_21994222*-qRT-F	AGGGTTGCAACCGTGGTAGCTC
*Cs_circ_21994222*-qRT-R	TTGACCCCCATGTTGCAAACC
*Cs_circ_67024327*-qRT-F	ACTCTTGCATGTGTTCTCGGTGG
*Cs_circ_67024327*-qRT-R	TTCAAGGCCAAGAAACTGCCC
*Cs_circ_13924653*-qRT-F	AAGGGTTGCCTGGTTGCATCA
*Cs_circ_13924653*-qRT-R	GAGTTTGCACGTGCTTCATCATCA
*Cs_circ_44508988*-qRT-F	GACAGGAGGTATTCCCAATGATTGG
*Cs_circ_44508988*-qRT-R	GCCTAACCATCTCGGTAAAGGCC
*Cs_circ_62648206*-qRT-F	TGGATGAGTCTTATCTGGGCAGCT
*Cs_circ_62648206*-qRT-R	CTGAGCAACCAGAGAGAAAGCCC
*Cs_circ_7359214*-qRT-F	CGCTCAGACAGTAGGTCCAAGGAG
*Cs_circ_7359214*-qRT-R	TGAAGATCCTCTTCCAGGGTTGC
*CsSPT5-*qRT*-*F	GTGCGATTGGTAAAGAGCGAGAAG
*CsSPT5-*qRT*-*R	ATGTGCTTCCTTGTCCGCTTCA
*CsEF1α*-qRT-F	TGTTTTGCACGGATCAGTTTG
*CsEF1α*-qRT-R	AATGCCGACCGCTACAGTTC

**Table 3:** Primers used in quantitative real-time PCR.

Name of primers	Sequence of primers (5’-3’)
*Cs_miR395a*-qRT-F	CGTGAAGTGTTTGGGGGAACTC
*Cs_miR395g*-qRT-F	CGTTGAAGTGTTTGGGGGAACTC
*CsEF1α*-qRT-F	TGTTTTGCACGGATCAGTTTG
*CsEF1α*-qRT-R	AATGCCGACCGCTACAGTTC

Relative expression levels were calculated using the 2^-ΔΔ^*^Ct^* method. Expression data were normalized against the industrial hemp housekeeping gene *EF1α* (Chaoqiong et al., 2018), which was used as the internal control. All qRT-PCR analyses were conducted with three biological replicates, and data were presented as mean ± SEM. Statistical analysis was conducted using GraphPad Prism 10 (GraphPad Software, La Jolla, CA, USA). Differences between the 6-BA treated and control groups were evaluated using one-way ANOVA followed by Duncan’s multiple range test, with *P* < 0.05 considered statistically significant.

## 3 Results

### 3.1 Identification and validation of circRNA back-splicing junctions in industrial hemp

**Figure 1:**
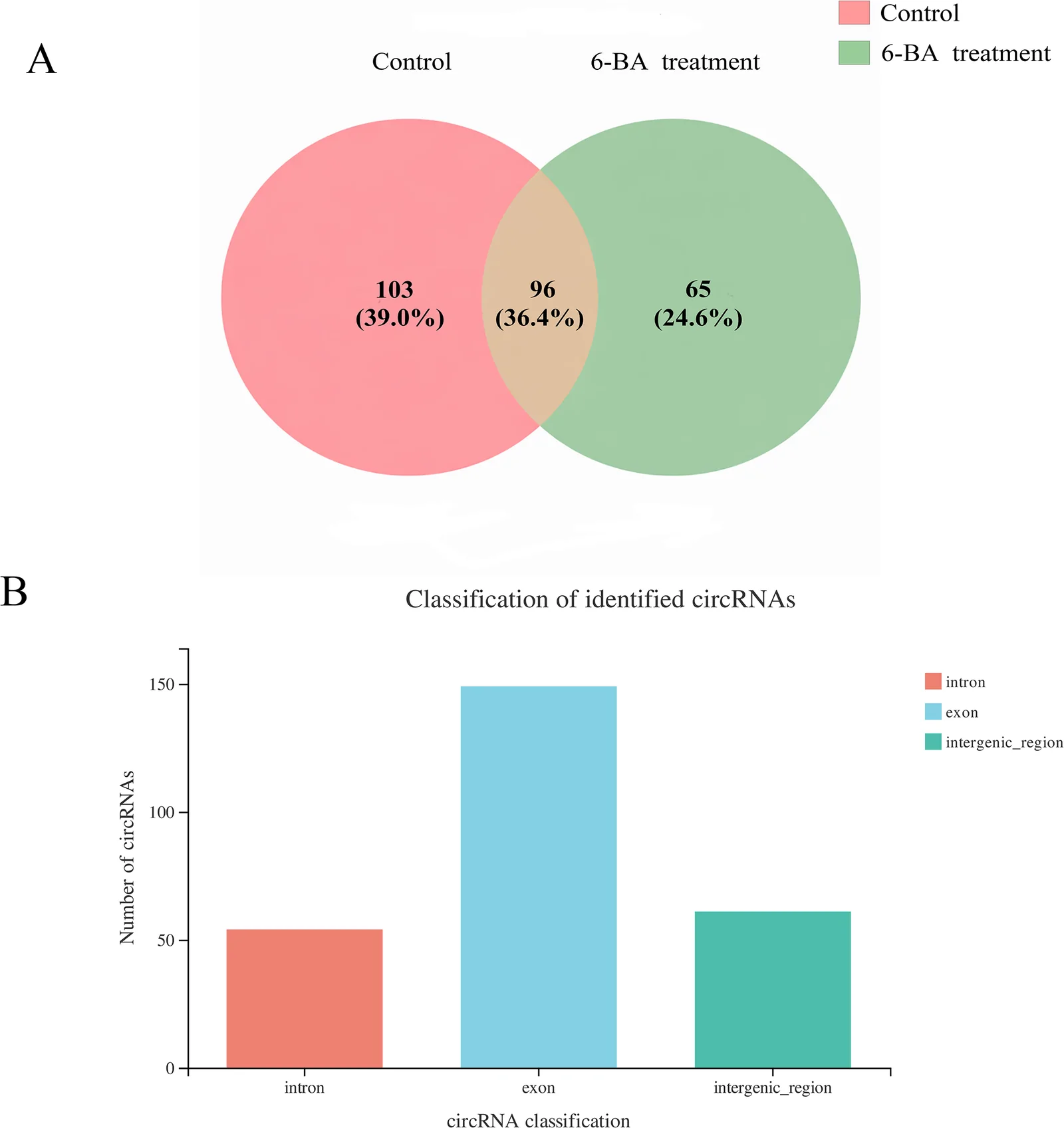
Characterization of circRNAs in industrial hemp. **(A)** Venn diagram showing the number and distribution of circRNAs detected in the control group and 6-BA treatment group. **(B)** Origin and distribution characteristics of circRNAs in industrial hemp. The number of circRNAs in each category is shown. The horizontal axis represents the classification of circRNAs, and the vertical axis represents the number of circRNAs.

To investigate the expression profile of circRNAs in industrial hemp under 6-BA treatment, RNA was isolated from female flowers of industrial hemp treated with 60 mg·L^-1^ 6-BA (**Figure 1A**). Among them, 103 circRNAs were exclusively detected in the control group, 65 were specific to the 6-BA treatment group, and 96 were shared between the two groups, indicating the existence of circRNAs potentially responsive to 6-BA treatment. Among the circRNAs identified in the transcriptomic database, 54 (20.5%) were derived from intronic regions, 149 (56.4%) from exonic regions, and 61 (23.1%) from intergenic regions (**Figure 1B**).

**Figure 2:**
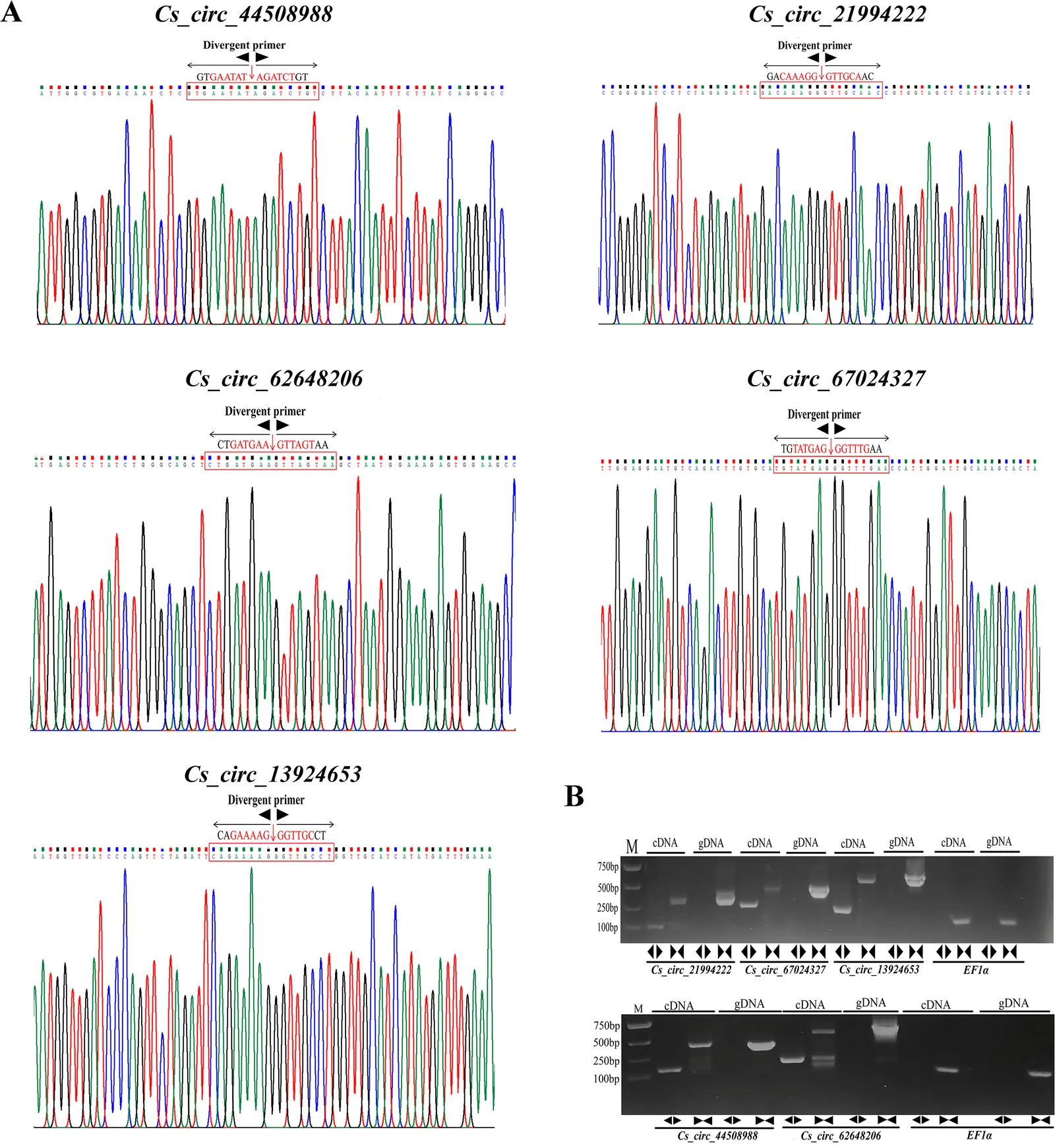
Validation strategy for circRNAs. **(A)** Sanger sequencing chromatograms confirming the back-splice junctions of five circRNAs. Red arrows indicate the junction sites. **(B)** Agarose gel electrophoresis of products amplified using divergent and convergent primers from cDNA and gDNA templates. Agarose gel electrophoresis showed that divergent primers amplified PCR products of the expected size in cDNA but failed to do so in genomic DNA (gDNA). Convergent primers successfully amplified products from both cDNA and gDNA. *EF1α* served as a linear control. Solid triangles represent convergent primers. Arrows represent divergent primers.

To validate the reliability of the identified circRNAs and explore their potential roles in floral sex differentiation, 19 representative candidate circRNAs were selected for experimental validation. Among these 19 circRNAs, 5 originated from introns, 11 from exons, and 3 from intergenic regions. To distinguish circRNAs from linear RNAs, we designed divergent primers and convergent primers, respectively (**Table 1**), and identified the amplification products by sanger sequencing (**Figure 2A**). Due to their covalently closed circular structure, the back-splicing junctions of circRNAs can only be specifically amplified using divergent primers with cDNA templates. As a control, genomic DNA (gDNA) was used as a template to verify that the back-splicing junction was absent from the genomic sequence, thereby confirming that the amplified product was a characteristic fragment of circRNAs. Through PCR validation and subsequent assays, the authenticity of the predicted back-splicing junctions of five circRNAs was successfully verified (**Supplementary Table 2**). Agarose gel electrophoresis and Sanger sequencing confirmed that the sequences of these back-splicing junctions were highly consistent with the RNA-seq results, strongly validating the genuine head-to-tail covalent linkage of these five circRNAs (**Figure 2B**). These findings clearly demonstrate the existence of RNA alternative circularization in industrial hemp, providing a reliable experimental basis for subsequent circRNA functional characterization.

### 3.2 Expression pattern analysis of circRNAs in industrial hemp under 6-BA treatment

**Figure 3:**
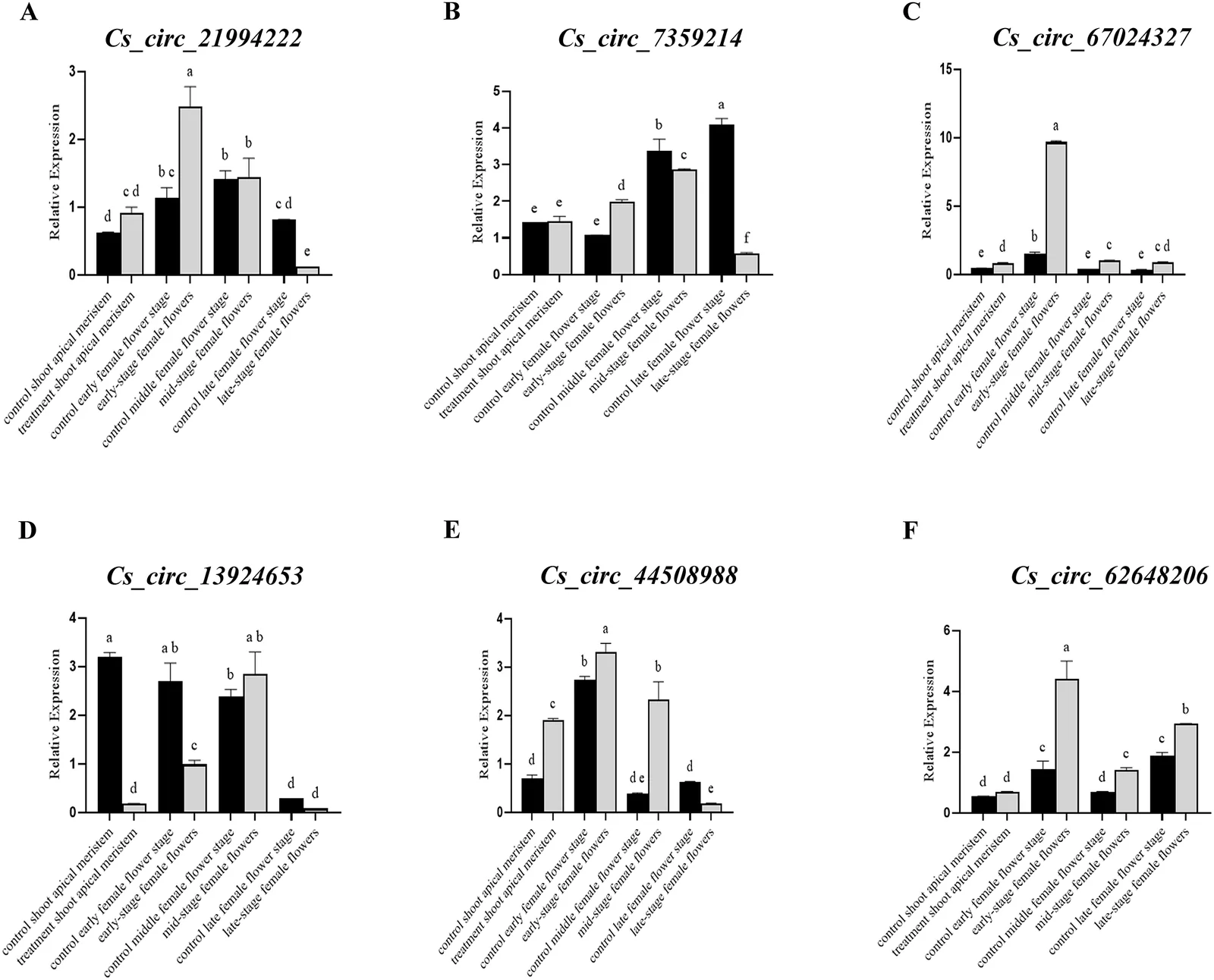
Validation of the expression levels of DECs by real-time quantitative PCR. The six randomly selected DECs are **(A)** Cs_circ_21994222, **(B)** Cs_circ_7359214, **(C)** Cs_circ_67024327, **(D)** Cs_circ_13924653, **(E)** Cs_circ_44508988, and **(F)** Cs_circ_62648206. Relative expression levels were determined in the shoot apical meristem (SAM) and at the early, middle, and late stages of female flower development under control and 6-BA treatment conditions. Black columns represent the control group, and gray columns represent the 6-BA treatment group. Data are shown as mean ± SEM from three independent biological replicates. Different lowercase letters above the columns indicate statistically significant differences among groups (one-way ANOVA followed by Duncan’s multiple range test, P < 0.05).

To investigate the expression levels of circRNAs in industrial hemp under 6-BA treatment, six randomly selected circRNAs were quantified by qRT-PCR. The list of circRNAs selected for this study is detailed (**Table 2**). The qRT-PCR results demonstrated that all six selected circRNAs exhibited significant spatiotemporal expression specificity and distinct responsiveness to 6-BA during female flower development in industrial hemp (**Figure 3**). *Cs_circ_21994222*, *Cs_circ_67024327*, *Cs_circ_44508988*, and *Cs_circ_62648206* were significantly upregulated in early-stage female flowers under 6-BA treatment, and their expression levels showed varying degrees of downregulation at subsequent developmental stages, indicating that these four circRNAs are mainly involved in the early responses to 6-BA and may play key roles in the initial floral differentiation process triggered by 6-BA. For instance, *Cs_circ_62648206* reached its peak expression in 6-BA-treated early-stage female flowers and maintained high expression levels throughout later stages, suggesting its involvement not only in the early response but also in subsequent floral development. Notably, *Cs_circ_67024327* exhibited the most pronounced induction in 6-BA-treated early-stage female flowers, with an average relative expression of approximately 9.67, which was 6.48 times higher than that of the corresponding control group. These findings suggest that *Cs_circ_67024327* may be one of the most sensitive circRNAs in response to 6-BA treatment during the early phase of female flower differentiation. *Cs_circ_13924653* exhibited a unique expression profile. In the control group, it maintained relatively high expression levels in both the SAM and early/mid-stage female flowers. Under 6-BA treatment, its expression was downregulated in the SAM and early-stage female flowers, but recovered at the mid-stage of female flowers under 6-BA treatment. This suggests that *Cs_circ_13924653* is more closely involved in mid-stage regulatory processes, particularly in development after the initiation of female flower differentiation and in the maintenance of female characteristics.

Collectively, qRT-PCR validation confirmed the differential expression patterns of the selected circRNAs, suggesting that DECs may participate in distinct developmental stages of 6-BA-mediated female flower differentiation in industrial hemp.

**Figure 4:**
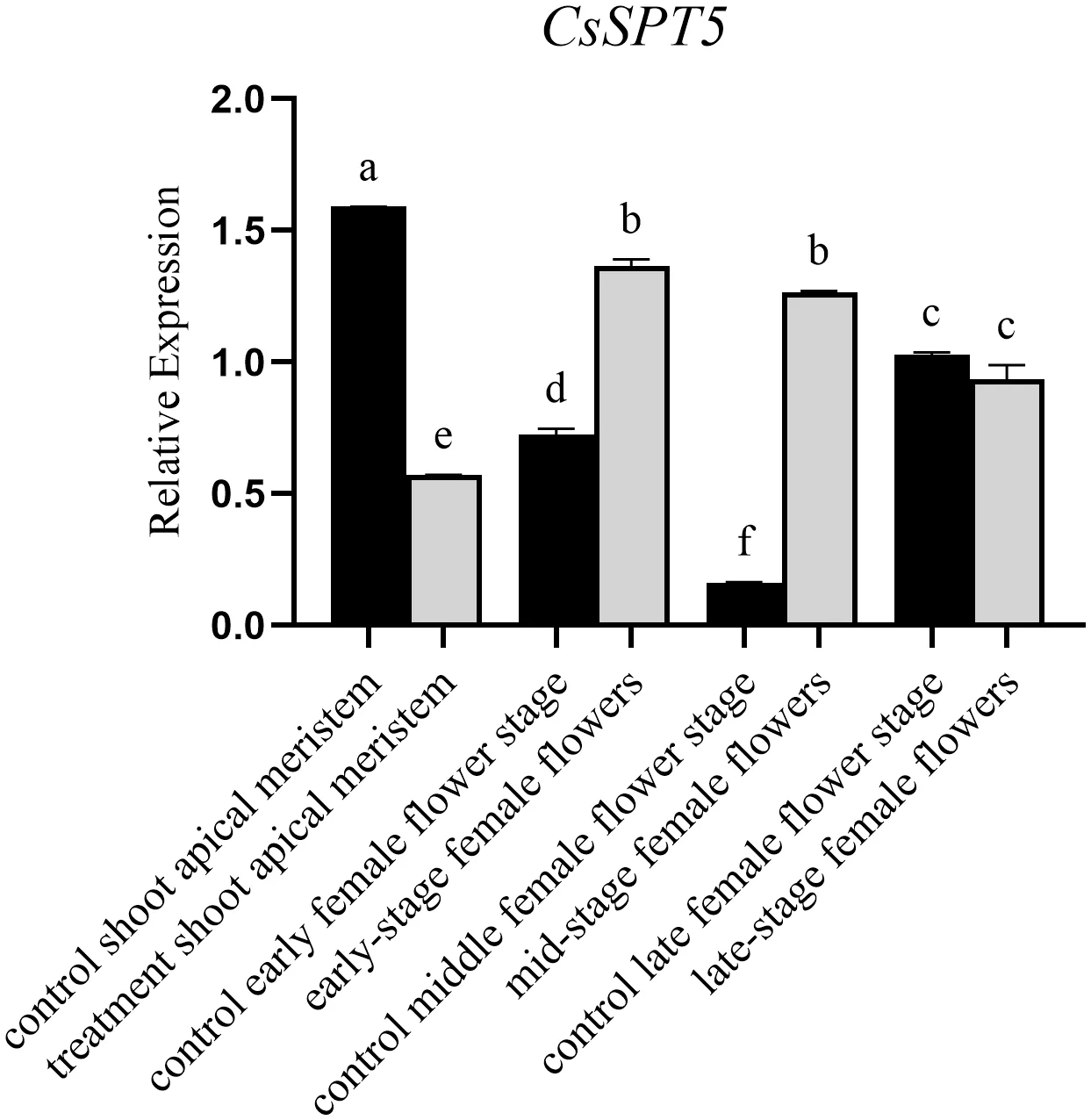
Validation of the expression levels of *CsSPT5* by real-time quantitative PCR. The qRT-PCR validation of *CsSPT5* during female flower development in industrial hemp under 6-BA treatment. The relative expression levels were determined in the SAM and at the early, middle, and late stages of female flower development under both control and 6-BA treatment conditions. Black columns represent the control group, and gray columns represent the 6-BA treatment group. Data are shown as mean ± SEM based on three independent biological replicates. Different lowercase letters above the columns indicate statistically significant differences among all groups (one-way ANOVA followed by Duncan’s multiple range test, *P* < 0.05).

A recent pangenome analysis has revealed that the flowering-time gene *SPT5* resides near the SDR/PAR boundary of cannabis, and its differential expression may affect the stability of sex differentiation (Lynch et al., 2025). To further evaluate the potential association between the 6-BA-responsive circRNA module and the *CsSPT5*-related pathway, we examined the expression patterns of *CsSPT5* across different developmental stages of female flowers. The results demonstrated that the response of *CsSPT5* to 6-BA exhibited distinct stage specificity (**Figure 4**). Under natural control condition, *CsSPT5* expression exhibited its highest level at the SAM stage, decreased at the early female flower stage, dropped to the lowest level at the mid female flower stage, and rebounded at the late female flower stage. Compared with that in the control group at the corresponding stage, *CsSPT5* expression was significantly downregulated by 6-BA treatment at the SAM stage, but markedly upregulated at both the early and mid female flower stages, with the most pronounced induction observed at the mid-stage. No significant difference in *CsSPT5* expression was detected between the treatment and control groups at the late female flower stage. These findings indicate that *CsSPT5* does not continuously respond to 6-BA throughout the entire process of female flower development; instead, it shows a prominent response mainly during the initiation and progression of female flower differentiation.

### 3.3 Functional enrichment analysis of circRNA host genes and miRNA target genes under 6-BA treatment

To investigate the potential biological roles of host genes associated with DECs and target genes of DEMs under 6-BA treatment, both sets of genes were subjected to GO functional annotation and KEGG pathway enrichment analysis. After Benjamini-Hochberg multiple testing correction (with an adjusted *P*-value threshold<0.05), neither gene set exhibited statistically significant enrichment of GO terms or KEGG pathways; only nominal enrichment trends were observed based on uncorrected *P*-values. For DEC host genes, nominally enriched terms primarily involved external stimulus response, defense response, phosphate starvation response, and metabolic processes. For DEM target genes, nominal enriched terms were associated with cellular components and biological processes including the CCAAT-binding factor complex, transcriptional regulatory complexes, root meristem growth, and cytokinin signaling. Taken together, these results should be interpreted as exploratory functional trends rather than statistically significant enrichment findings. Detailed enrichment results are presented in **Supplementary Tables 3**–**S6**.

### 3.4 Prediction of circRNA-miRNA-mRNA regulatory networks

**Figure 5:**
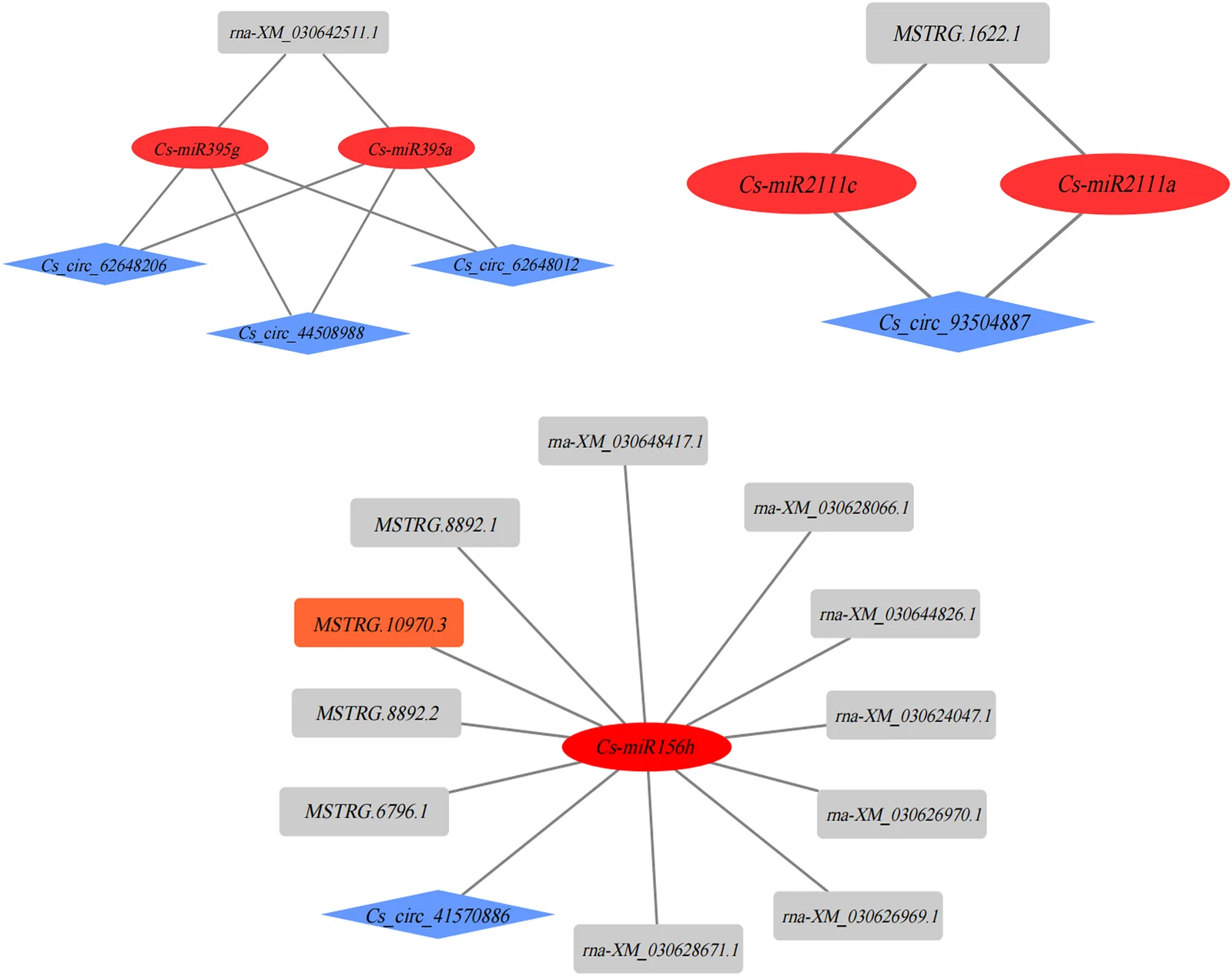
Schematic diagram of the putative circRNA-miRNA-mRNA interaction network in industrial hemp under 6-BA treatment. Diamond, circular, and rectangular nodes represent circRNAs, miRNAs, and mRNAs, respectively. Orange rectangular nodes denote mRNAs with reported or putative associations with sex differentiation, while gray rectangular nodes represent other mRNAs. Edges indicate bioinformatically predicted interactions rather than experimentally validated regulatory relationships.

Recent studies have demonstrated that circRNAs act as miRNA sponges and regulate gene expression, thereby playing important roles in the post-transcriptional regulation of downstream target genes. To investigate whether industrial hemp circRNAs influence the post-transcriptional levels of target genes by binding to miRNAs, we employed bioinformatics approaches to identify potential miRNA binding sites within industrial hemp circRNAs (**Supplementary Table 7**). In this study, six circRNAs were identified to harbor potential miRNA binding sites, among which two were predicted to function as miRNA sponges (**Supplementary Table 8**). Since *Cs_miR166k-5p* had no predicted target mRNAs, we predicted the interactions between the other five circRNAs and these five miRNAs. The putative circRNA-miRNA-mRNA interaction network, constructed based on bioinformatic predictions and visualized with Cytoscape, showed that a single miRNA may be targeted by multiple circRNAs, while a single circRNA may target miRNAs. For example, *Cs_miR395a* and *Cs_miR395g* were predicted to be targeted by three circRNAs, whereas *Cs_circ_93504887* in industrial hemp was shown to target two miRNAs. In the prediction network for *Cs_circ_41570886*-*miR156h*-mRNA, *Cs_circ_41570886* was predicted to function as a molecular sponge for *Cs-miR156h*, which in turn regulates several candidate genes potentially associated with sex differentiation, including two *Arabidopsis* homologs: a leucine-rich repeat receptor-like serine/threonine-protein kinase (At1g56140) and squamosa promoter-binding-like protein 13A (SPL13A) (**Figure 5**). In addition, several proteins encoded by the predicted target mRNAs have been reported to be associated with plant reproductive development and sex differentiation, including probable alpha, alpha-trehalose-phosphate synthase (UDP-forming) 10 (TPS10), scarecrow-like protein 22, ethylene-responsive transcription factor RAP2-7, and the transcription factors NF-YA3 and NF-YA1. These results, derived from bioinformatic predictions and functional annotations, provide a reference for screening candidate regulators potentially involved in the regulation of sex differentiation in industrial hemp and lay the foundation for subsequent functional studies (**Table 4**).

**Table 4:** Functional characterization of miRNA target genes associated with plant sexual differentiation under 6-BA treatment.

miRNA name	Target RNA id	Host gene description
Cs_miR156h	MSTRG.10970.3	squamosa promoter-binding-like protein 13A, transcript variant X2
Cs_miR171-3p	rna-XM_030633390.1	scarecrow-like protein 22
Cs_miR171a		
Cs_miR171d-3p		
Cs_miR171e		
Cs_miR171e-3p		
Cs_miR171f		
Cs_miR171h		
Cs_miR171i		
Cs_miR171u		
Cs_miR172	rna-XM_030654323.1	probable alpha,alpha-trehalose-phosphate synthase [UDP-forming] 11
	MSTRG.26257.4	ethylene-responsive transcription factor RAP2-7, transcript variant X2
	MSTRG.26257.3	ethylene-responsive transcription factor RAP2-7, transcript variant X2
Cs_miR172b	rna-XM_030654323.1	probable alpha,alpha-trehalose-phosphate synthase [UDP-forming] 11
	MSTRG.26257.3 MSTRG.26257.4	ethylene-responsive transcription factor RAP2-7, transcript variant X2
Cs_miR172c	MSTRG.26257.4 MSTRG.26257.3	
Cs_miR172c-3p	MSTRG.26257.3	
	MSTRG.26257.4	
Cs_miR172d	MSTRG.26257.3 MSTRG.26257.4	
Cs_miR172d-3p	MSTRG.26257.4	
	MSTRG.26257.3	
Cs_miR172e	MSTRG.26257.4 MSTRG.26257.3	
Cs_miR172e-3p	MSTRG.26257.4 MSTRG.26257.3	
Cs_miR172g	MSTRG.26257.3	
	MSTRG.26257.4	
Cs_miR172k	MSTRG.26257.3	
	MSTRG.26257.4	
Cs_miR172j	MSTRG.26257.3	
	MSTRG.26257.4	
Cs_miR172i-3p	MSTRG.26257.4	
	MSTRG.26257.3	
Cs_miR172l	MSTRG.26257.4	
	MSTRG.26257.3	
Cs_miR169m	MSTRG.9393.13	nuclear transcription factor Y subunit A-3, transcript variant X11
Cs_miR169g	rna-XM_030639725.1	nuclear transcription factor Y subunit A-1, transcript variant X2
Cs_miR169v	rna-XM_030639725.1	nuclear transcription factor Y subunit A-1, transcript variant X2
Cs_miR390	rna-XM_030645636.1	LRR receptor-like serine/threonine-protein kinase ERL1, transcript variant X1
Cs_miR390.2		
Cs_miR390-5p		
Cs_miR390a		
Cs_miR390a-5p		
Cs_miR390b		
Cs_miR390b-5p		
Cs_miR390c		
Cs_miR390d		
Cs_miR390d-5p		
Cs_miR390f		

One miRNA may target multiple transcript variants of the same gene; all predicted target transcripts are listed separately to show the complete regulatory relationships.

**Figure 6:**
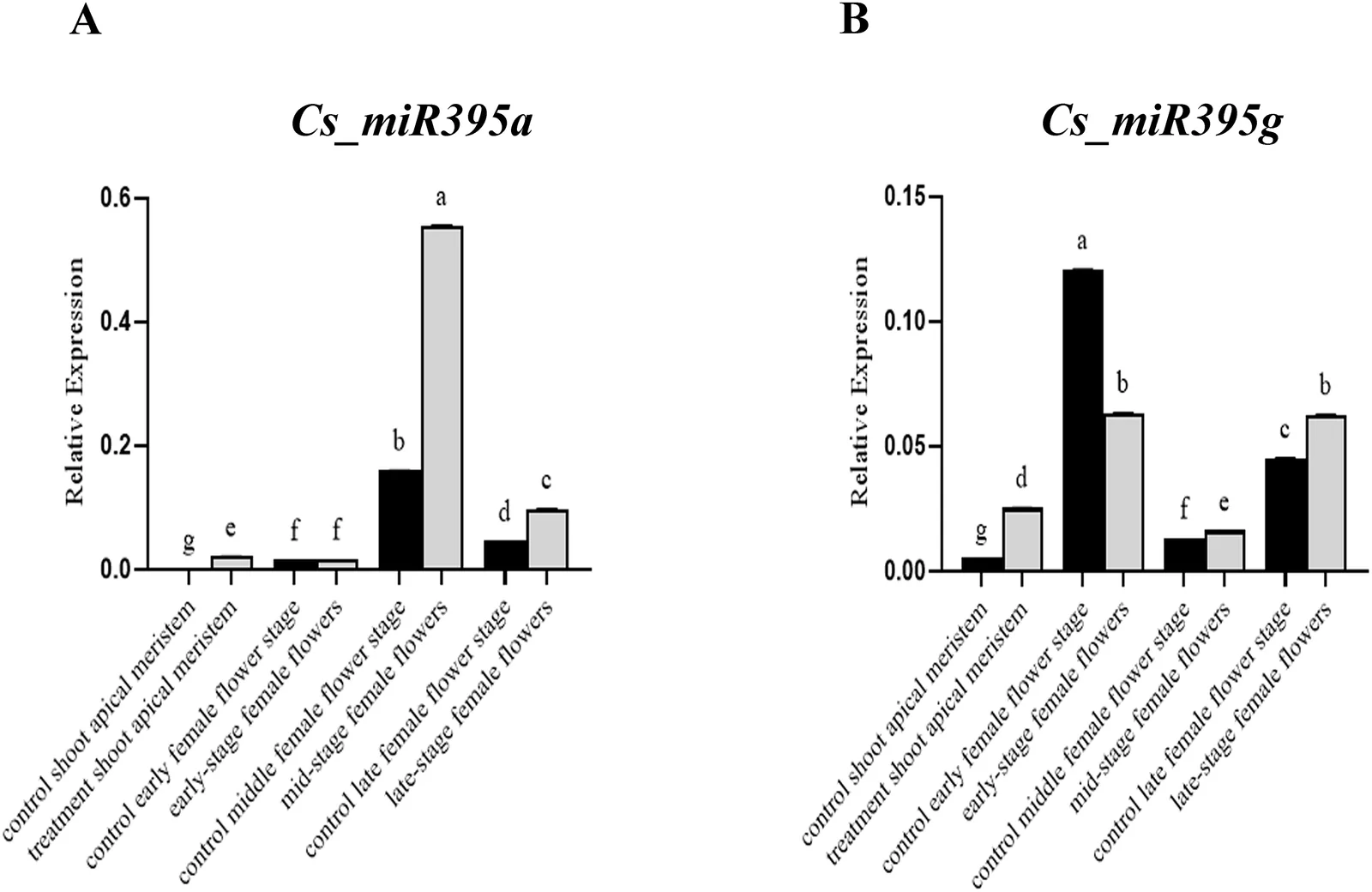
Validation of the expression levels of circRNA-targeted miRNAs by real-time quantitative PCR: **(A)** Cs_miR395a and **(B)** Cs_miR395g. Relative expression levels were measured in the shoot apical meristem (SAM) and at the early, middle, and late stages of female flower development in the control and 6-BA-treated groups. Black bars represent the control group, and gray bars represent the 6-BA-treated group. Data are shown as mean ± SEM from three independent biological replicates. Different lowercase letters above the bars indicate statistically significant differences among groups (one-way ANOVA followed by Duncan’s multiple range test, P < 0.05).

To verify whether 6-BA treatment significantly alters gene expression profiles, we compared the expression levels of target miRNAs between the control and 6-BA treatment groups at each developmental stage of female flowers. As shown in **Figure 6**, in the control group, *Cs_miR395a* maintained an extremely low basal expression level in the SAM and at the early female flower stage. Its expression then increased significantly, peaking at the middle female flower stage, and declining slightly at the late female flower stage. Under natural conditions, this miRNA mainly functions at the middle stage of female flower development, where it is involved in floral morphogenesis and physiological regulation. After 6-BA treatment, the expression of *Cs_miR395a* in the SAM was significantly upregulated compared with the control, indicating that 6-BA induced the expression of this miRNA in advance at the meristem stage in industrial hemp. At the early female flower stage, no significant difference was observed between the treatment and the control groups, indicating that the inductive effect had not yet emerged. In contrast, its expression was significantly upregulated at the middle female flower stage, which represented the stage with the strongest inductive effect of 6-BA. At the late female flower stage, its expression level remained significantly higher than that of the contemporaneous control, indicating a sustained inductive effect. For *Cs_miR395g*, its expression was extremely low in the SAM under control condition, rose sharply to peak at the early female flower stage, dropped rapidly to a low level at the middle female flower stage, and rebounded slightly at the late stage. Under natural condition, this miRNA primarily functions at the early stage of female flower development and may participate in the initiation of female floral differentiation. Upon 6-BA treatment, consistent with *Cs_miR395a*, the expression of *Cs_miR395g* in the SAM was significantly upregulated relative to the control. At the early female flower stage, however, its expression was significantly downregulated compared with the control, showing an inhibitory effect opposite to the response pattern of *Cs_miR395a*. No significant difference was observed between the two groups at the middle female flower stage, as both maintained low expression levels. At the late stage, its expression was slightly upregulated compared with the control.

**Figure 7:**
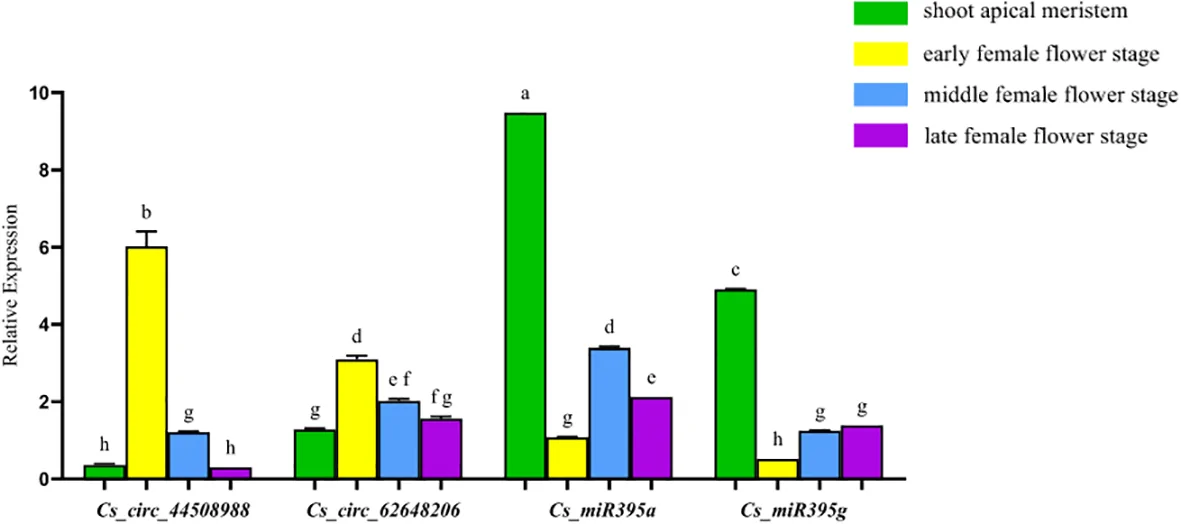
Relative expression profiles of four candidate regulators at different developmental stages of female flowers in industrial hemp. The detected regulators include two circRNAs (*Cs_circ_44508988*, *Cs_circ_62648206*) and two miRNAs (*Cs_miR395a*, *Cs_miR395g*). Green, yellow, blue and purple columns correspond to the SAM, early-, middle- and late-female flower stage, respectively. Data are expressed as mean ± SEM based on three independent biological replicates. Different lowercase letters above the bars indicate statistically significant differences among all groups (one-way ANOVA followed by Duncan’s multiple range test, *P* < 0.05).

Meanwhile, to investigate the regulatory interaction between circRNAs and miRNAs, we performed qRT-PCR analysis of *Cs_circ_44508988*, *Cs_circ_62648206*, *Cs_miR395a* and *Cs_miR395g* in the SAM and female flowers of industrial hemp. Bioinformatic target prediction indicated that both *Cs_circ_44508988* and *Cs_circ_62648206* harbor putative binding sites for *Cs_miR395a* and *Cs_miR395g*. The expression of *Cs_circ_44508988* peaked at the early stage of female flower development, with extremely low expression in the SAM and a rapid decline at the middle and late stages. Although *Cs_circ_62648206* showed a milder decline at the middle and late stages, its expression also peaked at the early female flower stage. In contrast, *Cs_miR395a* exhibited the highest expression level in the SAM, decreased to the lowest level at the early female flower stage, and rebounded moderately at the middle stage. Likewise, *Cs_miR395g* also showed the highest expression in the SAM, dropped to the minimum at the early female flower stage, and gradually recovered during the middle and late stages. Its overall expression trend was consistent with that of *Cs_miR395a*, but with a weaker rebound at the middle stage. These results demonstrate that circRNAs and miRNAs generally exhibit opposite expression trends, and the expression levels of these two circRNAs are negatively correlated with those of *Cs_miR395a* and *Cs_miR395g* (**Figure 7**). These findings are largely consistent with the predicted ceRNA regulatory model, suggesting that the relevant circRNAs may have potential regulatory interactions with miRNAs and their downstream target genes. Further studies are required to characterize the functions of these candidate molecules and elucidate their specific roles in the regulation of sex differentiation within the purely predictive network of industrial hemp.

### 3.5 Prediction of the protein-coding potential of industrial hemp circRNAs under 6-BA treatment

Studies have demonstrated that circRNAs have the capability to directly recruit ribosomes and initiate translation via internal ribosome entry site (IRES) elements. To evalute the protein-coding potential of industrial hemp circRNAs under 6-BA treatment, the IRES finder website (http://iresite.org/) was used to predict potential IRES elements within the circRNA sequences. Meanwhile, the ORF finder (https://www.ncbi.nlm.nih.gov/orffinder/) was used to identify complete ORFs within circRNAs and assess their coding potential. The results revealed that four DECs each contained at least one IRES element and harbored one or more open reading frames (ORFs) (**Supplementary Tables 9**, **10**). To further elucidate the functional characteristics of circRNAs, we performed conserved domain analysis on these five circRNAs via the online platform at the website (https://www.ncbi.nlm.nih.gov/Structure/cdd/wrpsb.cgi). In addition, conserved domain analysis was conducted on the conserved structural regions of the five identified circRNAs using the Conserved Domain Database (CDD), aiming to assess the evolutionary conservation of their coding regions. Notably, highly conserved regions are more likely to possess protein-coding potential (**Supplementary Table 11**).

The results showed that only *Cs_circ_3988107* was predicted to encode a calcium-binding protein, belonging to the EF-hand superfamily. Among the previously identified circRNAs, *Cs_circ_21994222* and *Cs_circ_44508988* were found to contain conserved domains corres- ponding to the Peptidase M1 aminopeptidase family and leucine-rich repeat receptor-like protein kinase (LRR-RLK), respectively. *Cs_circ_21994222* was predicted to encode a core region encompassing the catalytic domain of the M1 family of aminopeptidases, which exhibits a conserved pattern of continuous or partial repeats within this domain. *MPA1* (Meiotic Prophase Aminopeptidase 1), a member of the Peptidase M1 aminopeptidase-like protein family in *Arabidopsis thaliana*, has been shown to regulate cell cycle progression during meiosis in male and female gametophytes (Matsui et al., 2006). Therefore, Peptidase M1 is predicted to act as a potential indirect regulator in plant sex differentiation. If a specific member of this family targets key proteins or peptides within sex-determination pathways, it could serve as an important regulatory node in plant sex differentiation. The circRNA *Cs_circ_44508988* is predicted to encode a conserved domain of LRR-RLK, and its putative protein product may retain LRR-RLK activity. LRR-RLKs have been demonstrated to regulate male and female gametophyte development, function as a signaling hub in plant sex differentiation, and participate in various processes including plant growth, development, and stress responses (Lee et al., 2024). *Cs_circ_3988107* was predicted to encode a calcium-binding protein belonging to the EF-hand superfamily. Calcium plays a critical role in pollen development and pollen tube growth. In rice, *OsDEX1* mutants exhibit disrupted calcium homeostasis, leading to delayed tapetum degeneration and subsequent impairment of pollen formation. Calcium-binding proteins such as *OsDEX1* are involved in regulating tapetum cell death, callose degradation and pollen exine formation. Disruption of calcium homeostasis results in tapetal dysfunction and arrested pollen development, ultimately leading to male sterility (Yu et al., 2016). These findings suggest that circRNA-encoded proteins may contribute to the regulation of sex differentiation in industrial hemp under 6-BA treatment.

## 4 Discussion

CircRNAs are covalently closed non-coding RNAs that were initially regarded as nonfunctional byproducts of splicing errors (Barrett and Salzman, 2016). However, with advance in technologies such as high-throughput sequencing, an increasing number of circRNAs have been identified in various plants species. Accumulating evidence indicates that circRNAs perform vital functions in multiple biological processes, including growth and development (Zhang et al., 2020), hormonal regulation (Bhar and Roy, 2023), and stress responses (Litholdo and da Fonseca, 2018). CircRNAs have been systematically identified in several plant species, such as *Arabidopsis thaliana* (Chen et al., 2017), rice (Zhou et al., 2021b), tomato (Fan et al., 2020), and seabuckthorn (Zhang et al., 2019), and relevant investigations have also been reported in industrial hemp (Liu et al., 2023). Previous studies have demonstrated that 60 mg·L^-1^ 6-BA exerts a significant feminizing effect on industrial hemp (Yu et al., 2024). Therefore, in this study, industrial hemp plants were treated with 60 mg·L^-1^ 6-BA and circRNAs associated with female flower development were identified using high-throughput sequencing and bioinformatic analyses to clarify their functions and characteristics. Differential expression analysis was performed to screen circRNAs with specifically high expression or differentially expression in female flowers, and key circRNAs related to female flower differentiation were identified. We further explored the downstream sex differentiation associated miRNAs of these circRNAs and analyzed the circRNA-miRNA-mRNA regulatory networks, with the aim of uncovering the molecular regulatory mechanisms underlying feminization in industrial hemp.

### 4.1 Preferential production of exonic circRNAs under 6-BA treatment

Using female industrial hemp flowers treated with 60 mg·L^-1^ 6-BA and back-splice junction sequencing, we identified 264 circRNAs (**Figure 1A**), providing valuable resources for the construction of an industrial hemp circRNA database. Analysis of their genomic origins showed that exonic circRNAs were the most abundant (149, 56.4%), followed by intergenic circRNAs (61, 23.1%), while intronic circRNAs accounted for the lowest proportion (54, 20.5%) (**Figure 1B**). This distribution pattern is consistent with those reported in *Arabidopsis thaliana* and tomato (Meng et al., 2018; Yang et al., 2020), as well as with previous findings on non-coding RNAs associated with cannabinoid biosynthesis (Liu et al., 2023).

### 4.2 Stage-specific expression of circRNAs and the *miR395* family members in 6-BA-mediated female flower differentiation

Accumulating evidence indicates that certain circRNAs are implicated in plant sex differentiation. In citrus, 176 DECs were identified, many of which interact with genes regulating floral organ development and sex determination (Zeng et al., 2018). Similarly, in pecan, 46 DECs identified during flower bud differentiation were involved in flowering regulation, suggesting that circRNAs play specific roles in regulating plant sex differentiation (Jin et al., 2023). Our qRT-PCR validation demonstrated that both the DECs and members of the *miR395* family exhibited prominent developmental stage-specific expression patterns in response to 6-BA, suggesting that they exert hierarchical regulatory roles in 6-BA-mediated female flower differentiation in industrial hemp.

These dynamic expression changes indicate that circRNAs do not exert uniform effects throughout the entire developmental process; instead, they participate in distinct regulatory stages of female flower differentiation. Under 6-BA treatment, four circRNAs (*Cs_circ_67024327*, *Cs_circ_21994222*, *Cs_circ_44508988* and *Cs_circ_62648206*) were significantly upregulated at the early stage of female flower development, exhibiting typical characteristics of early-responsive regulators. Among them, *Cs_circ_67024327* showed the highest expression level across all detected circRNAs and all tested developmental stages, indicating that its high sensitivity to 6-BA during the early phase of female flower differentiation. The high abundance of these four circRNAs at the early stage suggests their potential involvement in the transition of the SAM from vegetative maintenance to female reproductive development. Notably, they exhibited distinct spatiotemporal patterns after early induction: the expression of *Cs_circ_21994222* declined sharply from the middle to late stage, suggesting its function is largely restricted to the early response phase of female flower development; *Cs_circ_44508988* maintained high expression at the early and middle stages but decreased significantly at the late stage, indicating that it primarily functions during the early and middle phases; *Cs_circ_62648206* maintained high expression even at the late stage under 6-BA treatment, implying its involvement in both early induction and late-stage female flower development. In contrast, *Cs_circ_13924653* displayed a unique expression profile: although its expression was downregulated in the SAM and early-stage female flowers upon 6-BA treatment, it recovered to relatively high levels at the middle developmental stage. This pattern indicates that *Cs_circ_13924653* is more closely associated with regulatory events downstream of female flower differentiation initiation, and may contribute to maintaining or promoting the establishment of female floral traits. As for *Cs_circ_7359214*, its expression accumulated progressively with flower development in the control group, but was significantly suppressed at the late stage under 6-BA treatment, suggesting that 6-BA mainly acts on the late developmental maturation process mediated by this circRNA rather than affecting the differentiation initiation stage.

In addition to circRNAs, the two members of *miR395* family also exhibited clear functional divergence and differential responses to 6-BA during female flower development. Although both miRNAs belong to the *miR395* family, they showed distinct temporal expression patterns and differential response profiles to 6-BA across female flower development. In untreated industrial hemp plants, *Cs_miR395a* was significantly upregulated at the middle stage of female flower development, while *Cs_miR395g* was markedly elevated at the early stage. Notably, at the early female flower stage, 6-BA had no significant effect on *Cs_miR395a* but significantly repressed the expression of *Cs_miR395g* at the same stage. Both miRNAs shared a common response pattern: they were upregulated by 6-BA in the SAM and slightly induced at the late stage of female flower development. Collectively, the stage-specific expression patterns of these non-coding RNAs provide potential targets for in-depth dissection of the regulatory network underlying 6-BA-mediated female flower differentiation in industrial hemp.

### 4.3 A circRNA-miRNA module may underlie feminization

CircRNAs can function as molecular sponges for miRNAs, sequestering miRNAs and thereby preventing their binding to target mRNAs, which in turn suppresses miRNA-mediated gene silencing (Valinezhad Orang et al., 2014). In rice, CRISPR-Cas9-generated circRNA deletion mutants have revealed that *Os06circ02797* sequesters *OsMIR408*, blocking *miR408*-dependent regulation of its target genes and consequently affecting seedling growth and chlorophyll accumulation, indicating a potential miRNA-sponge role of plant circRNAs (Zhou et al., 2021a). To date, a total of 741 circRNAs have been identified and characterized in industrial hemp. Under NaHCO_3_ stress, miRNA–mRNA regulatory network analysis has identified 230 interaction pairs involving 16 miRNAs and 179 target mRNAs (Cao et al., 2023). In this study, among the six analyzed circRNAs, one contained a potential binding site for a single miRNA, three harbored two miRNA binding sites (**Supplementary Table 8**), and one contained multiple binding sites targeting the same miRNA family, specifically the miR2111 family. This finding is consistent with a previous report in industrial hemp (Hasan et al., 2016). Although there is no direct evidence linking TPS10 to plant sex differentiation, studies in *Arabidopsis thaliana* have shown that loss of function of trehalose-6-phosphate synthase 1 (a homolog of AtTPS11) prevents plants from entering the floral transition stage (Dijken et al., 2004). Therefore, TPS10 may be indirectly involved in the regulation of flowering in plants. *SCL22* and *HvSCL* are members of the *SCL6* subfamily within the *GRAS* gene family. In barley, overexpression of *miR171* downregulates *HvSCL*, leading to extended vegetative growth, delayed flowering, reduced tiller number, and abnormal inflorescence development. These results clearly indicate that *miR171* regulates plant development by targeting SCL family members (Curaba et al., 2013). Similarly, in this study, industrial hemp transcript *XM_030633390.1* was found to be targeted by the *miR171* family, consistent with observations in barley. As a member of *AP2/ERF* family, *RAP2–7* functions mainly in transcriptional regulation of the ethylene signaling pathway, consistent with the established role of the *ERF* subfamily in ethylene-mediated plant growth and development. *ERF* family members have been reported to influence flowering time and floral development in industrial hemp (Adal et al., 2021), but the involvement of *RAP2–7* in flowering regulation remains to be elucidated. The transcription factors *NF-YA3* and *NF-YA1* are also closely associated with the regulation of plant sex differentiation (Siriwardana et al., 2016). CircRNAs are bioinformatically predicted to contribute to sex differentiation in industrial hemp. To explore the expression relationship between circRNAs and their target miRNAs, we analyzed *Cs_circ_44508988* and *Cs_circ_62648206* and their corresponding target miRNAs (*Cs_miR395a* and *Cs_miR395g*). The results revealed a significant inverse expression correlation between circRNA and miRNA (**Figure 7**). Specifically, circRNA expression was highest at the early stage, when miRNA expression was at its minimum, whereas at the mid- and late stages, circRNA abundance decreased while miRNA expression was significantly upregulated. These observations support the ceRNA sponge model, in which circRNAs are predicted to suppress miRNA activity by sequestering them. During early female flower development, highly abundant circRNAs capture miRNAs, reducing their free levels and relieving the silencing of downstream target mRNAs, thus facilitating the initiation of female flowers and the differentiation of floral primordia. In the mid-to-late stages of female flower development, decreased circRNA expression weakens miRNA sequestration, leading to increased free miRNA concentrations and reinforcing repression of their target genes, which in turn modulates floral organ differentiation and female flower maturation. Both *Cs_circ_44508988* and *Cs_circ_62648206* target *Cs_miR395a* and *Cs_miR395g* and show highly similar expression trends, implying that they function cooperatively as miRNA sponges during industrial hemp female flower development.

Collectively, combined with spatiotemporal expression profiles and functional annotation results, *Cs_circ_44508988*, *Cs_circ_62648206* and *miR395* family may form stage-specific ceRNA regulatory relationships, which can be categorized into two core regulatory axes. The first is the early initiation regulatory axis: at the early stage of female flower development, the two circRNAs are induced by 6-BA and sequester *Cs_miR395g* via molecular sponge activity, thereby inhibiting its function and facilitating the initiation of female flower differentiation. The second is the middle-stage homeostasis regulatory axis: at the middle stage, these two circRNAs interact with *Cs_miR395a* to buffer its expression fluctuations triggered by 6-BA, ensuring stable progression of female flower morphogenesis.

In terms of functional division, *Cs_circ_44508988* acts mainly at the early stage and shows strong temporal specificity. In contrast, *Cs_circ_62648206* exerts regulatory effects throughout the full developmental cycle of female flowers (early, middle and late stages), serving primarily as a sustained homeostatic regulator.

This circRNA-miRNA module likely represents a critical hub in the temporal regulation of industrial hemp female flower development. High circRNA expression in the early stage sequesters miRNAs, maintaining the expression of genes required for the initiation of female flower development. Subsequent downregulation of circRNA releases active miRNA, which direct floral organ differentiation and maturation, thereby ensuring proper morphogenesis and reproductive competence. Currently, no evidence supports a regulatory role for transcript *XM_030642511.1* in plant sex differentiation. However, the regulatory interaction between *Cs_circ_44508988*, *Cs_circ_62648206*, and *Cs_miR395a*/*Cs_miR395g* may serve as a key mechanism promoting female differentiation in industrial hemp. Future studies should investigate the spatiotemporal expression patterns of these miRNA target genes, including transcript *XM_030642511.1*, across different stages of female flower development in industrial hemp.

### 4.4 Protein-coding potential expands the functional repertoire of circRNAs

Research has demonstrated that circRNAs containing ORFs can be translated through IRESs, suggesting that some circRNAs possess protein-coding potential (Prats et al., 2020). In silico analysis using predictive software revealed that four circRNAs (*Cs_circ_38710397*, *Cs_circ_30304404*, *Cs_circ_91735295* and *Cs_circ_3988107*) each contain at least two IRES elements and an ORF (**Supplementary Table 10**), implying their capacity to encode proteins. Additionally, *Cs_circ_3988107*, *Cs_circ_21994222* and *Cs_circ_44508988* were predicted to encode a CML protein, an M1 family aminopeptidase and an LRR-RLK, respectively (**Supplementary Table 11**). It has been reported that numerous *CML* genes are essential for pollen development and pollen tube growth (Wang et al., 2008; Chen et al., 2009; Zhou et al., 2009). For instance, loss-of-function mutants in *CML24* and *CML25* in *Arabidopsis* lead to severely impaired pollen germination and pollen tube elongation (Wang et al., 2015; Yang et al., 2014). As noted above, Peptidase M1 family aminopeptidases are associated with plant sex differentiation. A key family member, *MPA1*, is essential for normal meiosis and gamete fertility-processes that determine sexual phenotype and form the foundational basis of sex differentiation, especially in dioecious species. Male sterility represents a widespread mechanism underlying the development of female flowers or plants in many species. Complete pollen sterility converts flowers into functionally female structures, while female sterility tends to promote male floral traits. Consequently, genes that regulate gamete fertility, such as *MPA1*, may play a critical role in determining sex ratios and the development of unisexual flowers (Peer, 2011). The qRT-PCR analysis revealed that *Cs_circ_21994222* was highly expressed during the early stage. Since this circRNA is predicted to encode a Peptidase M1 family aminopeptidase, it is necessary to further investigate its potential role in industrial hemp in sex differentiation and its involvement in promoting female flower development. As a circRNA predicted to encode an LRR-RLK receptor kinase, *Cs_circ_44508988* may function similarly to *Arabidopsis EMS1.* In *Arabidopsis*, *EMS1*-associated male-sterile mutants show excessive microsporocyte, tapetum loss, and abnormal persistence of middle-layer cells. While nuclear meiosis proceeds normally, defective cytokinesis leads to microspore abortion. Molecular characterization has shown that the *EMS1* gene encodes an LRR-RLK, and its expression is closely associated with the differentiation of germ cells and somatic cells in anthers (Zhao et al., 2002). This study demonstrates that LRR-RLKs are directly involved in regulating plant sex differentiation through modulation of cell fate determination in anthers. Our qRT-PCR analysis demonstrates that the expression level of *Cs_circ_44508988* is relatively high in the early stages. As it is predicted to encode an LRR-RLK, 6-BA treatment may specifically induce LRR-RLK-encoding genes via this circRNA. CircRNAs may exert their regulatory effects by targeting one or more LRR-RLK genes. By sponging *Cs_miR395*, these circRNAs relieve the repressive effect on downstream target transcripts (e.g., RNA *XM_030645636.1*), leading to enhanced LRR-RLK expression in floral primordia. These LRR-RLKs then sense and transduce signals that promote female development or inhibit male traits, thereby modulating downstream transcriptional cascades. Collectively, these findings indicate that circRNA-encoded proteins may participate in the regulation of sex differentiation towards femaleness in industrial hemp in response to 6-BA treatment.

### 4.5 The expression pattern of *CsSPT5* exhibits a stage-specific correlation with the 6-BA-responsive circRNA module

Recent genome-wide studies have revealed that the sex determination region (SDR) of industrial hemp contains a large set of genes associated with agronomic traits and flowering time. The *SPT5* gene, located near the SDR-PAR boundary, has been characterized as a candidate regulator of flowering time and sex chromosome evolution (Lynch et al., 2025). Given its conserved function in *FLC/FRIGIDA*-mediated flowering regulation in *Arabidopsis*, *SPT5* may serve as an important molecular link between sex chromosome organization and floral development timing in industrial hemp. To further investigate whether *CsSPT5* is involved in 6-BA-induced female flower differentiation in a stage-dependent manner, we examined its expression patterns across four developmental stages: the SAM, early female flower stage, mid female flower stage, and late female flower stage. qRT-PCR results demonstrated that the response of *CsSPT5* to 6-BA treatment exhibited pronounced developmental stage specificity. Rather than continuously responding to hormonal signals throughout the entire course of female flower development, it displayed strong responsiveness primarily during the initiation and progression of female flower differentiation. This stage-specific expression pattern showed partial temporal overlap with the expression dynamics of a subset of 6-BA-responsive circRNAs. Multiple circRNAs identified in this study also displayed distinct stage-specific expression patterns during 6-BA-induced female flower development. Specifically, *Cs_circ_67024327* was strongly induced at the early female flower stage, suggesting its potential involvement in the early signaling response to 6-BA-triggered female flower differentiation. The expression levels of *Cs_circ_44508988* and *Cs_circ_62648206* increased significantly at the early female flower stage; although they declined to varying degrees at the mid-to-late stages, they remained at detectable expression levels, indicating that both mainly function in the initiation of female flower differentiation and the early-to-mid developmental stages. In addition, *Cs_circ_13924653* exhibited a unique expression rebound at the mid female flower stage, implying its potential role in mid-stage morphogenesis following the initiation of female flower differentiation. Notably, *CsSPT5* was significantly upregulated by 6-BA at both the early and mid stages of female flower development, which closely coincided with the core response window of the aforementioned circRNAs. This finding indicated that *CsSPT5* and the 6-BA-responsive circRNA module might jointly regulate the stage transition of female flower development.

Integrating the expression profiles of *CsSPT5* and the 6-BA-responsive circRNAs, we speculate that upon 6-BA treatment, *CsSPT5* expression was initially suppressed at the SAM stage, while most circRNAs had not yet reached their expression peaks. As female flower development proceeded to the early and mid stages, *CsSPT5* was significantly induced and upregulated, accompanied by stage-dependent expression fluctuations in multiple 6-BA-responsive circRNAs, including *Cs_circ_67024327*, *Cs_circ_44508988*, *Cs_circ_21994222*, and *Cs_circ_62648206*. Accordingly, we propose that 6-BA may drive the stage-wise progression of female flower differentiation at both the transcriptional and post-transcriptional levels by simultaneously modulating the flowering time-related factor *CsSPT5* and the non-coding RNA regulatory network. Among these candidates, *Cs_circ_44508988* may serve as a core regulatory node linking 6-BA signaling, miRNA-mediated post-transcriptional regulation, and the expression of downstream flower development-related genes. In future studies, the potential regulatory relationship between *CsSPT5* and *Cs_circ_44508988* will be further validated, and the expression differences of candidate circRNA-miRNA-mRNA regulatory modules between dioecious and monoecious cannabis cultivars will be systematically dissected to elucidate the coupling mechanism between the non-coding RNA network and the canonical sex determination pathway.

In this study, 264 circRNAs of industrial hemp were identified and their back-splice junction was experimentally verified. Multiple circRNAs were confirmed to respond to 6-BA treatment, among which *Cs_circ_67024327* is the most prominent early-responsive member. The response of *CsSPT5* to 6-BA treatment also exhibits distinct developmental stage specificity. Functional enrichment analyses indicated that circRNA host genes are associated with stress responses, metabolic regulation, and DNA replication or repair-related processes, although no GO terms or KEGG pathways reached statistical significance after multiple testing correction. The opposite temporal expression patterns of the circRNAs and miR395a/g are compatible with the predicted ceRNA model, although this model remains to be experimentally validated. In addition, several circRNAs were predicted to possess protein-coding potential or conserved functional domains, suggesting that industrial hemp circRNAs may be involved in 6-BA-induced female differentiation through multiple putative regulatory mechanisms. In conclusion, this study provides preliminary correlative evidence for the involvement of 6-BA-responsive circRNAs in female flower differentiation of industrial hemp. The proposed ceRNA network and its associated coding potential remain hypothetical, and require further experimental validation in follow-up research.

## Data Availability

The raw RNA-seq data generated in this study have been deposited in the NCBI Sequence Read Archive (SRA) under BioProject accession number PRJNA1445027 and are publicly available at https://www.ncbi.nlm.nih.gov/bioproject/PRJNA1445027/.
